# The Association of SNPs Located in the CDKN2B-AS1 and LPA Genes With Carotid Artery Stenosis and Atherogenic Stroke

**DOI:** 10.3389/fneur.2019.01170

**Published:** 2019-11-22

**Authors:** Anetta Lasek-Bal, Dorota Kula, Tomasz Urbanek, Przemysław Puz, Jan Szymszal, Michał Jarzab, Monika Halczok, Renata Cyplinska, Wiesław Bal, Beata Łabuz-Roszak, Aleksandra Cieślik, Ilona Jasnos, Barbara Jarzab, Damian Ziaja

**Affiliations:** ^1^Department of Neurology, School of Health Sciences, Medical University of Silesia, Katowice, Poland; ^2^Maria Skłodowska-Curie, Memorial Cancer Center and Institute of Oncology, Gliwice, Poland; ^3^Department of General Surgery, Vascular Surgery, Angiology and Phlebology, Medical University of Silesia, Katowice, Poland; ^4^Faculty of Technical Sciences, University of Occupational Safety Management in Katowice, Katowice, Poland; ^5^3rd Department of Radiotherapy and Chemotherapy, Maria Sklodowska-Curie Institute—Oncology Center, Gliwice, Poland; ^6^Department of Outpatient Chemotherapy, Maria Skłodowska-Curie, Memorial Cancer Center and Institute of Oncology, Gliwice, Poland; ^7^Department of Basic Medical Sciences, Faculty of Public Health, Medical University of Silesia, Katowice, Poland

**Keywords:** stroke, CDKN2B GENE, LPA gene, atherosclerosis, polymorphism

## Abstract

**Introduction:** The aim of this project was to assess the prevalence of four selected SNPs rs4977574 and rs7857345 (CDKN2B-AS1 gene) and rs3798220 and rs10455872 polymorphisms (the LPA gene) in the subpopulation of patients with symptomatic and asymptomatic carotid stenosis.

**Material and Methods:** This study included 623 individuals (244 patients with symptomatic carotid artery stenosis, 176 patients with asymptomatic carotid artery stenosis and 203 healthy people. All the participants underwent neurological examination, duplex Doppler ultrasound examination and molecular procedures.

**Results:** In the first part of the analysis the assiociation of SNPs with stroke/TIA was investigated. The association was seen in symptomatic vs. control group for two SNPs: rs4977574 and rs7857345 (CDKN2B-AS1 gene); genotype distributions for rs4977574 and rs7857345 showed the statistically significant differences between patients and controls (*p* = 0.043 and 0.017, respectively). No association was observed for rs3798220 and rs10455872 located in the LPA gene. There were statistically significant differences between asymptomatic patients vs. control group in genotype distribution for the SNPs located in CDKN2B-AS1: rs4977574 and rs7857345 (*p* = 0.031 and 0.0099, respectively); and for the rs3798220 (LPA gene; *p* = 0.003); however, statistically significant differences did not occur for the rs10455872 polymorphism located in the LPA gene. In the next part of the evaluation, a comparison between symptomatic and asymptomatic patients was performed. Significant differences in genotype distribution were seen only for the rs3798220 polymorphism located in the LPA gene (*p* = 0.0015). The analysis of the prevalence of the polymorphisms in the total group (symptomatic and asymptomatic) patients in comparison with the control group showed significant differences for three polymorphisms: rs4977574 and rs7857345 (CDKN2B-AS1 gene; *p* = 0.015 and 0.0046, respectively) and rs3798220 (LPA gene, *p* = 0.044).

**Conclusions:** The present research on the carotid artery stenosis patient cohort suggests the significant association between the rs4977574, rs7857345 and rs3798220 polymorphisms and carotid artery stenosis as well as between the rs4977574 and rs7857345 polymorphisms and atherogenic stroke. The rs4977574 and rs7857345 polymorphisms in patients with carotid artery stenosis appear to affect a person's susceptibility to atherogenic brain ischemia. Our results need to be replicated in future studies.

## Introduction

Atherosclerotic stenosis of carotid and/or cerebral arteries is responsible for ~30% of ischemic stroke cases. It was demonstrated that the risk of stroke, including a serious risk of cerebral ischemia in patients with a >60% stenosis of arterial lumen, rose proportionately to the increase in internal carotid artery stenosis. The factors that further modify the risk of stroke in that group of patients include the type of atherosclerotic plaque, the parameters of its stability, the coexistence of other diseases, and complex genetic and environmental relations. The basic method to assess the genetic factors for diseases is to evaluate the relationship between a disease entity and polymorphic gene variants, mainly single-nucleotide polymorphisms (SNPs). Previous studies on the link between specific genes and stroke demonstrated inconsistent results (1–3). Racial/ethnic differences in study subpopulations, differences in the protocol, and small research groups could have all contributed to those discrepancies. A possible reason could also be the non-selectivity of the subpopulation of patients with stroke. Considering pathophysiology and etiology, stroke is a heterogeneous disease (4). It is therefore likely that individual phenotypes of the disease may depend on the expression of various genes. The significance of the gene–stroke relationship is indicated in as much as 40% of patients with acute cerebral ischemia (3). Certainly, the role of genetic factors is variable and subject to the modifying role of the environment and traditional risk factors for stroke.

Non-specific genetic and etiological correlation was demonstrated mainly for cardio-embolic stroke and, to a lesser extent, for large-vessel and small-vessel disease (5–13). The contribution of genetics to stroke risk and whether this differs for different stroke subtypes remains uncertain. Previous candidate gene studies (GWAS) have identified many associations with stroke but whether these are important requires replication in large independent data sets. GWAS datasets provide a powerful resource to perform replication studies. So far, ~40 SNPs were shown as potentially associated with the risk of stroke. No previously reported candidate gene was significant following rigorous correction for multiple testing.

Therefore, there is a need for more studies on the relationship with various subtypes of acute cerebral ischemia. As of today, the relationship between specific polymorphisms and the risk of non-symptomatic carotid stenosis has not been studied. It seems that such research could contribute to a better understanding of the genetic basis of the disease in question.

The aim of this project was to assess the prevalence of four selected SNPs in the subpopulation of patients with symptomatic (stroke, transient ischemic attack; TIA) and non-symptomatic atherosclerotic carotid stenosis. According to the available literature overview in the study, the rs4977574 and rs7857345 polymorphisms located in the CDKN2B-AS1 gene as well as the rs3798220 and rs10455872 polymorphisms located in the LPA gene were selected (1, 14–20). An additional aim was to assess the co-occurrence of the abovementioned SNPs with other conditions/diseases recognized as traditional risk factors for stroke.

## Materials and Methods

### Subjects and Samples

This study included 623 individuals, including 244 patients with symptomatic carotid artery stenosis, 176 patients with non-symptomatic carotid artery stenosis, and 203 healthy people in the control group. The group of symptomatic patients included patients with common carotid artery stenosis and/or internal carotid artery stenosis (>60%) and with ischemic stroke and/or TIA occurring ipsilaterally to the stenotic carotid artery within the last 6 months. The group of non-symptomatic patients included those with common and/or internal carotid artery stenosis without a history of acute cerebral ischemia. Patients with atrial fibrillation (based on patient history and at least one 24-h ECG examination) and those with stroke of a different etiology than atherosclerosis (as per ASCOD) were not eligible for the study (4). The patients were recruited from among those hospitalized in the Departments of Neurology, General and Vascular Surgery of the Clinical Hospital No. 7 of Medical University of Silesia (in the period: 01.01.2016–01.01.2017). The control group included healthy individuals who reported to the clinic for consultation.

Each participant expressed their informed consent. The study was accepted by the Ethics Committee of the Silesian Medical University of Silesia in Katowice.

All the patients underwent neurological examination, duplex Doppler ultrasound examination of the carotid arteries performed with 5–7.5 MHz probe in search for stenotic plaque, and hemodynamically significant stenosis. Bilateral CCA and ICA assessments were performed with a possible stenosis measurement based on the NASCET criteria and velocity assessment. Patients with arterial (thrombotic or atherosclerotic) occlusion were excluded from the study. Plaque morphology was also evaluated according to the five classes proposed by Gray-Weale.

### DNA Extraction and SNP Analysis

DNA was isolated from whole blood by anion exchange membrane column separation (Genomic Maxi AX Blood; A&A Biotechnology, Gdynia, Poland) as per the manufacturer's protocol. Initial screening of DNA purity was determined based on the evaluation of optical density ratio at 260/280 nm (samples with a ratio of 1.8–2.0 were considered to be high purity).

SNPs (rs4977574 and rs7857345 located in the CDKN2B-AS1 gene, and rs3798220 and rs10455872 located in the LPA gene) were analyzed with allelic discrimination technique. The analysis was performed on 384-well plates (Applied Biosystems, Foster City, CA, USA) using the 7900HT Fast Real-Time PCR System (Applied Biosystems, Foster City, CA, USA). Reactions were performed in the final volume of 5.1 μl per sample containing 0.125 μl of 40 × TaqMan® SNP Genotyping Assay (Applied Biosystems, Foster City, CA, USA), 2.5 μl of 2 × TaqMan Genotyping Master Mix (Applied Biosystems, Foster City, CA, USA), and 2.5 μl of DNA (at concentrations of 3 ng/μl).

### Statistical Analysis

The first analysis considered the presence of the selected four SNPs in the subpopulation of symptomatic (stroke, TIA) and non-symptomatic atherosclerotic carotid artery stenosis, and in the control group. The odds ratios (ORs) were calculated for the studied alleles using Wolf's formula. Deviations from the Hardy–Weinberg equilibrium for each polymorphism were verified. A *p* < 0.05 was considered to be statistically significant.

Next, separate analyses were applied to the group of symptomatic patients and the group of non-symptomatic subjects; the relationship between polymorphisms and risk factors for stroke were analyzed, i.e., age, sex, non-atrial fibrillation disturbances of heart rhythm, arterial hypertension, coronary artery disease, lipid disorders, diabetes mellitus, cigarette smoking, alcohol abuse, family stroke burden, chronic kidney disease, valvular disease, and cardiomyopathy.

Statistical calculations were performed using the following statistics packages: Statistical v. 7.1 PL by StatSoft and MedCalc Statistical Software v. 17.6 (MedCalc Software bvba, Ostend, Belgium).

The Shapiro-Wilk test was used to verify compliance with distribution of a feature measured on a numerical scale (e.g., age); then, parametric Student's *t*-test was used to compare the levels of the studied feature in the case of compliance with normal distribution; the Mann–Whitney *U*-test (Wilcoxon Rank Sum Test) was used if the distribution deviated from normal.

The chi-square test was used to verify the hypothesis of the independence of two features measured on a qualitative (nominal) scale by assessing the level of *p* using the maximum likelihood method.

Logistic regression was used to assess the influence of individual factors on the presence of genotypes by estimating the OR level, its confidence interval (CI), and the level of *p*. A statistical significance level of *p* < 0.05 was applied for statistical assessment.

## Results

**Figure 1:**
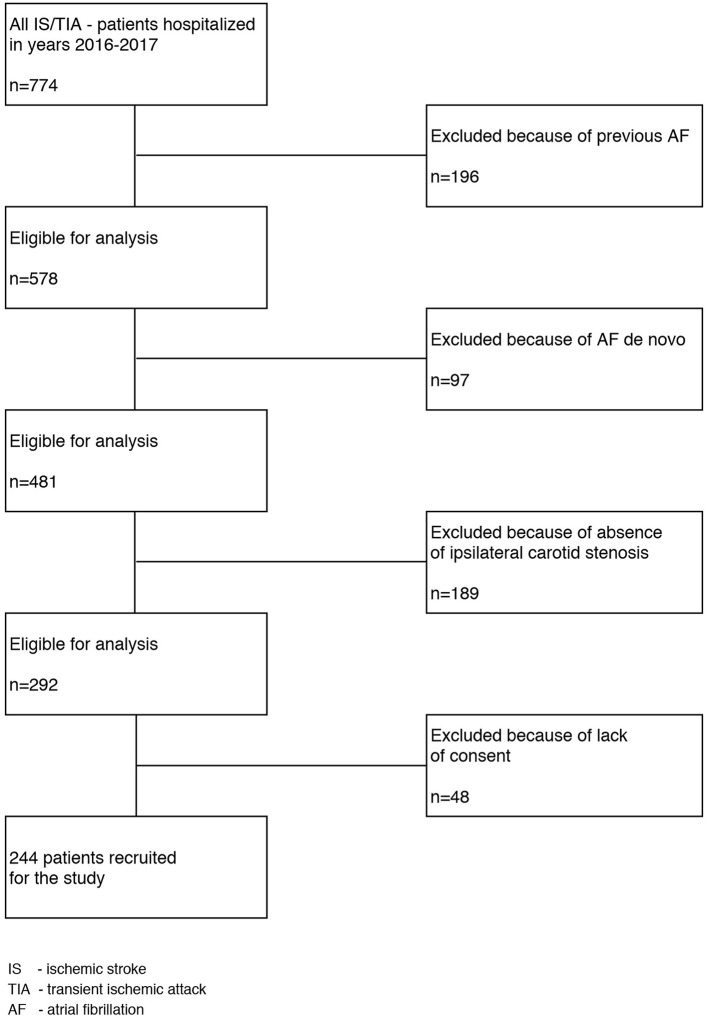
The flow chart of the patients' selection process. Is, ischemic stroke; TIA, Transient ischemic attack; AF, Arial fibrillation.

The recruitment scheme of stroke/TIA patients is shown in Figure 1.

Among 213 pre-qualified patients with asymptomatic carotid artery stenosis, ultimately 176 gave the informed consent to participate in this study.

Description of patients with symptomatic and non-symptomatic stenosis of the carotid artery is shown in Table 1.

**Table 1:** Characteristics of patients with symptomatic and non-symptomatic stenosis of the carotid artery.

**Parameter**		**SS; *n* = 244**	**NSS; *n* = 176**	***p***
**Age, year**	**mean; med. [min-max]**	70; 71 [29–96]	71; 71 [43–87]	0.9163
Sex (F)	N [%]	151 [61.88]	55 [31.25]	<0.0001
nAF	N [%]	10 [4.01]	10 [5.68]	0.3504
AH	N [%]	219 [89.75]	156 [88.64]	0.0548
CAD	N [%]	141 [57.79]	82 [46.59]	0.2001
MI past	N [%]	34 [13.93]	47 [26.70]	0.6484
LD	N [%]	111 [45.59]	137 [77.84]	<0.0001
DM	N [%]	102 [41.80]	31 [17.61]	<0.0001
O	N [%]	72 [29.51]	91 [51.70]	0.2625
CS	N [%]	111 [45.49]	96 [54.54]	0.0062
ALC	N [%]	29 [11.88]	15 [8.52]	0.0278
FSB	N [%]	31 [12.70]	8 [4.55]	0.0075
CKD	N [%]	29 [11.88]	32 [18.18]	0.0303
PAD	N [%]	32 [13.11]	35 [19.89]	0.0245
VD	N [%]	37 [15.16]	0 [0]	<0.0001
CM	N [%]	85 [34.83]	3 [1.7]	<0.0001
HF	N [%]	35 [14.34]	4 [2.27]	<0.0001

*SS, symptomatic stenosis group; NSS, non-symptomatic stenosis group; nAF, Non-atrial fibrillation disturbances of heart rhythm; AH, Arterial hypertension; CAD, Coronary artery disease; F, Female; MI past, Past myocardial infarction; N, Number of cases; LD, Lipid disorders; DM, Diabetes mellitus; O, Obesity; CS, Cigarette smoking; ALC, Alcohol abuse; FSB, Family stroke burden; CKD, Chronic kidney disease; PAD, Peripheral artery disease; VD, Valvular disease; CM, Cardiomyopathy; HF, Heart failure*.

The mean age of patients with symptomatic stenosis was 70.0 years [median 71.0 ± 8.6 (29–96)]; patients with asymptomatic stenosis: 70.1 years [median 71.0 ± 12.4 (43–87)] and control group: mean 50.3 years old [median 50.0 ± 16.74 (29–85)]. While the age difference between the patients and the control group was statistically significant (*p* < 0.0001), there was no statistical significance between the groups of symptomatic and non-symptomatic patients (*p* = 0.9163).

There were 113 (55.66%) women in the control group. All individuals from the control group were healthy, without known risk factors for cardiovascular disease, without chronic treatment, and were recruited from volunteers consulted to assess their health.

Comparison of the prevalence of genotypes for the analyzed polymorphisms in the groups of symptomatic patients, non-symptomatic patients, and control group are presented in Table 2.

**Table 2:** Comparison of the prevalence of genotypes for the analyzed polymorphisms in the groups of symptomatic patients, non-symptomatic patients, and control group.

**SNP Gene**	***N***	**Genotype**			***P*-value for genotype comparison**	**Type of comparison**	**Dominant model** **OR (95% CI); *p*-value**	**Recessive model** **OR (95% CI); *p*-value**
rs4977574 CDKN2B-AS1		**AA (%)**	**AG (%)**	**GG (%)**		G-risk allele	AG + GG vs. AA	GG vs. AG + AA
Symptomatic patients	235	61 (25.96)	112 (47.66)	62 (26.38)	**0.043**	Symptomatic patients vs. control	1.40 (0.92-2.11) 0.12	**1.76 (1.10-2.81); 0.017**
Non-symptomatic patients	171	45 (26.32)	78 (45.61)	48 (28.07)	**0.031**	Non-symptomatic patients vs. control	1.37 (0.87-2.15); 0.17	**1.92 (1.17-3.15) 0.0097**
					0.91	Symptomatic vs. non-symptomatic Patients	1.08 (0.69-1.68); 0.93	0.87 (0.56-1.35); 0.71
Patients in total	406	106 (26.11)	190 (46.80)	110 (27.09)	**0.015**	patients in total vs. control	1.38 (0.95-1.99); 0.08	**1.83 (1.19-2.80); 0.0055**
Control group	201	66 (32.84)	101 (50.25)	34 (16.92)				
rs7857345 CDKN2B-AS1		**CC (%)**	**CT (%)**	**TT (%)**		C-risk allele	CT + CC vs. TT	CC vs. CT + TT
Symptomatic patients	232	120 (51.72)	88 (37.93)	24 (10.34)	**0.017**	Symptomatic patients vs. control	1.63 (0.93-2.87); 0.09	**1.70 (1.16-2.50); 0.006**
Non-symptomatic patients	173	83 (47.98)	79 (45.66)	11 (6.36)	**0.0099**	Non-symptomatic patients vs. control	**2.77 (1.35-5.68); 0.0041**	1.47 (0.97-2.21); 0.07
					0.17	Symptomatic vs. non-symptomatic patients	0.59 (0.28-1.24); 0.16	1.16 (0.78-1.72); 0.46
Patients in total	405	203 (50.12)	167 (41.23)	35 (8.64)	**0.0046**	Patients in total vs. control	**1.99 (1.19-3.32); 0.0076**	**1.59 (1.13-2.25); 0.007**
Control group	202	78 (38.61)	92 (45.54)	32 (15.84)				
rs3798220 LPA		TT (%)	CT (%)	CC (%)		C-risk allele	CT + CC vs. TT	CC vs. CT + TT
Symptomatic patients	238	229 (96.22)	9 (3.78)	0 (0)	0.19	Symptomatic patients vs. control	0.57 (0.24-1.36); 0.20	0.85 (0.02-42.77); 0.93
Non-symptomatic patients	169	153 (90.53)	7 (4.14)	9 (5.33)	**0.003**	Non-symptomatic patients vs. control	1.51 (0.71-3.24); 0.28	**23.85 (1.38-412.96); 0.0029**
					**0.0015**	Symptomatic vs. non-symptomatic patients	**0.38 (0.17-0.87); 0.019**	**0.035 (0.002-0.61); 0.02**
Patients in total	407	382 (93.86)	16 (3.93)	9 (2.21)	**0.044**	Patients in total vs. control	0.94 (0.47-1.89); 0.88	9.6 (0.55-165.9); 0.11
Control group	201	188 (93.53)	13 (6.47)	0 (0)				
rs10455872 LPA		AA (%)	GA (%)	GG (%)		G-risk allele	GA + GG vs. TT	GG vs. GA + AA
Symptomatic patients	240	213 (88.75)	27 (11.25)	0 (0)	0.39	Symptomatic patients vs. control	1.23 (0.66-2.28); 0.52	0.28 (0.01-6.93); 0.43
Non-symptomatic patients	173	156 (90.17)	16 (9.25)	1 (0.58)	0.98	Non-symptomatic patients vs. control	1.06 (0.53-2.10); 0.88	1.17 (0.07-18.92; 0.91
					0.41	Symptomatic vs. non-symptomatic patients	1.16 (0.61-2.21); 0.64	0.24 (0.01-5.90); 0.38
Patients in total	413	369 (89.35)	43 (10.41)	1 (0.24)	0.73	Patients in total vs. control	1.16 (0.66-2.04); 0.62	0.49 (0.03-7.88); 0.62
Control group	203	184 (90.64)	18 (8.87)	1 (0.49)				

*N, number of cases; OR, odds ratio; CI, confidence interval. The bold values are statistically significant*.

An association of SNP with stroke was analyzed during the first stage of the study. Such an association was seen for two SNPs: rs4977574 and rs7857345 located in the CDKN2B-AS1 gene; genotype distributions for rs4977574 and rs7857345 showed some statistically significant differences between cases and controls (*p* = 0.043 and 0.017, respectively). No association was observed for rs3798220 and rs10455872 located in the LPA gene. During the next stage of analysis, OR values were calculated with an assumption of dominant or recessive inheritance models. For the rs4977574 polymorphism, an increased risk of the disease was observed for the GG genotype with a recessive model of inheritance (OR = 1.76; 95% CI: 1.10–2.81; *p* = 0.017). For the rs7857345 polymorphism, a significantly increased OR value was seen for the CC genotype (with a recessive model of inheritance) (OR = 1.70; 95% CI: 1.16–2.50; *p* = 0.006). OR values calculated for the rs3798220 and rs10455872 polymorphisms with dominant and recessive models of inheritance did not change significantly (OR = 1.23. *p* = 0.52 and OR = 0.28. *p* = 0.43, respectively).

When non-symptomatic patients were compared to control patients, statistically significant differences in genotype distribution were seen for the SNPs located in CDKN2B-AS1, that is, the rs4977574 and rs7857345 (*p* = 0.031 and 0.0099, respectively), and also for the rs3798220 polymorphism located in the LPA gene (*p* = 0.003); however, statistically significant differences did not occur for the rs10455872 polymorphism also located in the LPA gene (*p* = 0.98). Next, OR values were calculated with an assumption of dominant and recessive models of inheritance. Increased OR values were seen for the rs4977574 GG homozygotes (OR = 1.92, *p* = 0.0097, recessive model). Significantly increased OR values were observed for the rs7857345 polymorphism, as calculated with an assumption of a dominant model for the presence of C allele (OR = 2.77, *p* = 0.0041). For the rs3798220 CC homozygotes (recessive model), increased OR values were observed (OR = 23.85, *p* = 0.0029). The OR values calculated for the rs10455872 polymorphism with dominant and recessive models of inheritance did not change significantly (OR = 1.06, *p* = 0.88 and OR = 1.17, *p* = 0.91, respectively).

A comparison between symptomatic and non-symptomatic patients was performed during the next stage of the study. Significant differences in genotype distribution were seen only for the rs3798220 polymorphism located in the LPA gene (*p* = 0.0015). The next stage was to estimate the OR values based on the assumption of dominant and recessive models of inheritance. For the rs3798220 polymorphism, the observed protective effect of the C allele in the group of stroke/TIA patients vs. the group of patients without stroke/TIA was significant both with the dominant (OR = 0.38 *p* = 0.019) and the recessive model (OR = 0.035, *p* = 0.02).

During the next stage, we analyzed the prevalence of genotypes of the polymorphisms under study in the total group of (symptomatic and non-symptomatic) patients in comparison with the control group. The analysis showed significant differences for three polymorphisms: rs4977574 and rs7857345 (CDKN2B-AS1 gene; *p* = 0.015 and 0.0046, respectively) and rs3798220 (LPA gene *p* = 0.044). However, after calculating the OR, significant changes in the values were obtained only for the polymorphisms located in the CDKN2B-AS1 gene: increased OR (recessive inheritance model) was demonstrated (OR = 1.83, *p* = 0.0055) for homozygous GG carriers of the rs4977574 polymorphism; however, increased OR values were observed for the rs7857345 polymorphism in the presence of the C allele, both with the dominant (OR = 1.99, *p* = 0.0076) and the recessive model assumptions (OR = 1.59. *p* = 0.007).

### Association of SNPs With Other Stroke Risk Factors

During the first stage, multivariate logistic regression analysis concerning additional factors for stroke (listed in Table 1) was performed in the group of patients with stroke/TIA. The analysis showed a significant relationship between the rs4977574 GG homozygous polymorphism and cardiomyopathy, hypercholesterolemia, and circulatory failure. In GG homozygous carriers, there was an increased risk of cardiomyopathy (OR 1.96, 95% CI: 1.0038–3.84, *p* = 0.0487), while the risks of hypercholesterolemia and circulatory failure were reduced (OR = 0.43, 95% CI: 0.22–0.82, *p* = 0.01 and OR = 0.51; 95% CI: 0.27–0.96; *p* = 0.037, respectively). However, no significant clinical parameters were identified to be associated with the AG heterozygote or the AA homozygote. For the rs7857345 polymorphism, a significant correlation was found regarding family history: stroke/TIA patients who were CC homozygous carriers declared positive family history less frequently (i.e., stroke in family members) than patients with the CT and TT genotypes (OR = 0.33; 95% CI: 0.14–0.79; *p* = 0.01).

Then, multivariate logistic regression analysis was performed in the group of patients without stroke/TIA. For the rs4977574 polymorphism, GG homozygotes were shown to be associated with the age of onset; GG homozygotes were reported less frequently in older patients (OR = 0.96, 95% CI: 0.92–0.99; *p* = 0.048). The rs7857345 CC homozygous polymorphisms proved to be associated with cigarette smoking and alcohol consumption. CC homozygote carriers were more often smokers (OR = 2.06, 95% CI: 1.09–3.91, *p* = 0.026) but they consumed alcohol less frequently (OR = 0.26, 95% CI: 0.07–4.402, *p* = 0.87). The rs7857345 CT heterozygous polymorphism carriers showed positive family history more frequently (OR = 9.04, 95% CI: 1.09–75.16, *p* = 0.04). In patients with the TT variant, renal failure was significantly more frequent (OR = 6.58, 95% CI: 1.86–23.21, *p* = 0.003). An important factor demonstrating an association with the rs3798220 CC homozygous polymorphism was gender: the CC homozygotes were observed more frequently in women; men showed them less frequently (OR = 0.13, 95% CI: 0.025–0.66, *p* = 0.014); however, the TT variant was more frequent in men (OR = 5.51, 95% CI: 0.18–17.09, *p* = 0.003).

While searching for local factors related to plaque echogenicity and morphology between symptomatic and asymptomatic patients with significant stenosis of the carotid artery, no significant differences were seen according to the stenosis grade between the groups (*p* > 0.05). In both (symptomatic and asymptomatic) groups, a similar percentage of Gray-Weale plaque I + II and III + IV was found between the symptomatic and non-symptomatic patients (*p* > 0.05).

## Discussion

The existing results of the studies on the link between specific genes and stroke are inconsistent. We decided to assess the association of atherosclerotic carotid artery stenosis and atherogenic stroke/TIA with four polymorphic variants of the CDKN2B-AS1 and LPA genes. Our selection of genes for the study in a Polish subpopulation was guided by the literature overview and previous studies performed on the atherosclerotic patient populations (1, 14–20). We included patients with atherogenic stroke/TIA (a clinical manifestation of atherosclerosis of the arteries) and individuals with a hemodynamically significant stenosis of the artery without brain damage in order to determine if there are differences in the genetic variants in these two groups of patients.

We demonstrated that the rs4977574 and rs7857345 polymorphisms were significantly more frequent in patients with atherosclerotic asymptomatic carotid stenosis and complicated stroke/TIA than in healthy subjects. A higher risk of comorbid artery stenosis with stroke was observed for the rs4977574 GG and the rs7857345 CC homozygous polymorphism carriers, both under recessive model of inheritance. Stroke is a complex disease, and for most associations of common SNPs with common diseases, the genetic model of inheritance is unknown. It is common in the analysis of associations with rare susceptibility alleles to analyze data assuming a dominant model. However, usually a strong preference for one genetic model is unjustified as it is not known if the disease will be associated with one or two copies of the risk allele (21). Thus, it is necessary to test different models of inheritance—and this is why in our analysis dominant and recessive models were evaluated. An association of the above homozygous variants (rs4977574 GG and the rs7857345 CC) with other diseases/conditions and coexistence of the GG (rs4977574) variant with cardiomyopathy in patients with stroke/TIA are worth stressing. To date, based on several GWASes and replication studies, the presence of this SNP has been demonstrated to increase the risk of coronary heart disease, a disease developed in the course of atherosclerosis (22–25). The results of this study also suggest a genetic and etiological correlation between the SNP with non-symptomatic carotid artery atherosclerosis and with one complicated by stroke/TIA. Similar results related to the analysis of complex correlations between rs4977574 and atherogenic stroke/TIA were also presented by other researchers (8, 10–12). However, there were also results negating such a relationship (3).

Interestingly, in this study, regardless of the presence of arterial pathology, cardiomyopathy (a risk factor for myocardial infarction) was reported more frequently in the rs4977574 GG polymorphism carriers with symptomatic carotid artery stenosis. It is worth stressing that there are some common risk factors for heart disease (coronary heart disease, myocardial infarction, and cardiomyopathy) and stroke. The above cardiovascular phenotypes may have a common genetic variant. We may have proven that the presence of the rs4977574 polymorphism in patients with arterial stenosis, regardless of such complications, leads to stroke/TIA. According to neuro-epidemiological data, ~1/4 of the patients with stroke also have comorbid heart disease and atherosclerosis of the carotid artery, which is a potential risk of acute cerebral ischemia (26). The coexistence of these diseases may probably be due to the relationship of atherosclerosis and the gene resulting in possible ischemia of the heart and/or brain.

Similarly to the Swedish researchers, we found a connection between the rs7857345 polymorphism and the onset of stroke in the course of large artery atherosclerotic stenosis (16). The carriers of the rs7857345 CC polymorphism who did not undergo stroke more often declared using nicotine.

The rs3798220 polymorphism in this study was significantly more often found in patients with non-symptomatic stenosis than in healthy subjects. So far, it has been demonstrated that genetic variants in the rs3798220 SNP in the Lp(a) gene (LPA) correlate with elevated Lp(a) levels, but it has not been clearly established whether the SNP is a risk factor for coronary or carotid artery disease (27–30). The results of the PROCARDIS study suggest a strong relationship between polymorphism and coronary heart disease (29). Also interesting in the context of the study cited is a potential association between the rs3798220 polymorphism and atherogenic stroke.

Helgadottir et al. found a prognostic significance of the rs3798220 polymorphism for coronary heart disease and atherogenic stroke. However, those authors did not confirm a relationship between the polymorphism and intima-media thickness (IMT) (17). In our study, we found polymorphism to be present significantly more often in patients with non-symptomatic stenosis than in healthy patients. The results of this paper and the cited authors may suggest the importance of that polymorphism for the process of deposition of atherosclerotic plaque, but not necessarily for their activation. Atherosclerosis is a complex disease process with a number of crucial cellular contributors (endothelial, smooth muscle and immune cells) (31, 32). There are many hypotheses proposed to explain the mechanisms of the initiation, progression, and rupture of atherosclerotic plaque. Various epidemiological studies in families and twins have revealed a genetic component of stroke risk; the gene variants involved in these pathways of atherosclerosis could also contribute to cardiovascular disease risk.

The rs3798220 CC homozygous variant that increases the risk of stroke/TIA was significantly found more often in women, while TT was more frequently reported in men. There are known gender differences in the aspect of stroke type, the incidence, and the age of onset. It is seen more often and earlier in men who suffer from stroke mainly caused by large artery atherosclerosis and heart disease. The dominant role of risk factors important for the development of atherosclerosis and related to lifestyle has been emphasized (33). These observations are in concordance with the results regarding the rs3798220 polymorphism in this study.

Our study did not show any relationship between the rs10455872 polymorphism located in the LPA gene in patients with atherosclerotic carotid artery stenosis, non-symptomatic stenosis, and one complicated by stroke/TIA as compared with healthy subjects. The results obtained are inconsistent with previous work (17, 34). As the polymorphism was clearly unassociated with atherosclerotic stenosis of arteries, it was not included in the analysis along with other conditions/diseases considered as traditional risk factors for stroke.

The testing carried out, as shown in the results summary, revealed a relationship between two polymorphisms (rs4977574 and rs7857345) located in the CDKN2B-AS1 gene and symptomatic common and/or internal carotid artery stenosis. These polymorphisms, and additionally the one located in the LPA gene (rs3798220), also showed an association with non-symptomatic stenosis of the carotid artery. A comparison between patients with symptomatic artery stenosis and patients with non-symptomatic artery stenosis showed significant differences between these groups for the rs3798220 polymorphism located in the LPA gene. There seems to be a relationship between the rs4977574 and rs7857345 polymorphisms with stroke/TIA regardless of artery stenosis. An analysis of the genotype distribution in the entire group of symptomatic and non-symptomatic patients, as compared with the control group, showed significant differences for the following polymorphisms: rs3798220 (LPA gene), rs4977574, and rs7857345 (CDKN2B-AS1 gene), just like the comparison between non-symptomatic patients and the control group, which indicates an association of these polymorphisms with carotid artery stenosis. However, the OR analysis showed a significantly modified risk of disease only for polymorphisms rs4977574 and rs7857345 located in the CDKN2B-AS1 gene.

This study was innovative in that it demonstrated similarities in the genetic profile of patients with both symptomatic and non-symptomatic artery stenosis. Therefore, it seems that genetic variants in the CDKN2B-AS1 and LPA genes have an effect on the function of arterial walls and/or the atherosclerotic process. CDKN2B-AS1 gene is located within the CDKN2B-CDKN2A gene cluster at chromosome 9p21. The gene product is a functional RNA molecule that interacts with polycomb repressive complex-1 (PRC1) and−2 (PRC2), leading to epigenetic silencing of other genes in that cluster. The region is a significant genetic susceptibility locus for cardiovascular disease and has also been linked to a number of other pathologies including several cancers, intracranial aneurysm, type 2 diabetes, periodontitis, Alzheimer's disease, endometriosis, frailty in the elderly, and glaucoma (35). The LPA gene encodes apolipoprotein(a) [apo(a)], which is a component of lipoprotein(a) (35). The protein encoded by this gene is a serine proteinase that inhibits the activity of tissue-type plasminogen activator I. The encoded protein constitutes a substantial portion of lipoprotein(a) and is proteolytically cleaved, resulting in fragments that attach to atherosclerotic lesions and promote thrombogenesis. Elevated plasma levels of that protein are linked to atherosclerosis (36).

We also determined a potential significance of individual polymorphisms in correlation with a patient's present clinical (and traditional) risk factors for stroke. Cardiomyopathy was more common in the subpopulation with symptomatic arterial stenosis. It may be a factor strengthening the expression of genes related to atherosclerosis or just a parallel manifestation of arterial disease with a common genetic variant. Special attention should therefore be paid to patients with atherosclerotic stenosis and additional cardiomyopathy, even one that causes no clinically evident arrhythmias.

The abovementioned results showing the significance of certain genetic variants only in atherogenic stroke/TIA are in concordance with the trend presented in the literature related to risk factors for stroke. It has been suggested that strokes of various etiologies should be considered as different diseases. Different profiles of conventional risk factors, various genetic patient profiles, and differing prognoses support such viewpoint. Possibly, specific polymorphic gene variants play an etiological role in different types of atherosclerotic disease.

We are aware of the limitations of our study; the most important of them are: one-site study and uneven distribution between the numbers of symptomatic vs. asymptomatic patients.

## Conclusions

The present research on the carotid artery stenosis patient cohort suggests the significant association between the rs4977574, rs7857345, and rs3798220 polymorphisms and carotid artery stenosis as well as between the rs4977574 and rs7857345 polymorphisms and atherogenic stroke.

The rs4977574 and rs7857345 polymorphisms in patients with carotid artery stenosis appear to affect a person's susceptibility to atherogenic brain ischemia.

Our results need to be replicated in future studies.

## Data Availability Statement

This manuscript contains previously unpublished data. The name of the repository and accession number(s) are available on request.

## Ethics Statement

The studies involving human participants were reviewed and approved by Bioethics Committee, Medical University of Silesia. The patients/participants provided their written informed consent to participate in this study.

## Author Contributions

AL-B and JS: study design, data collection, statistical analysis, data interpretation, manuscript preparation, literature search, and funds collection. DK: study design, data collection, statistical analysis, data interpretation, manuscript preparation, and literature search. TU: study design, data collection, statistical analysis, data interpretation, literature search, and funds collection. PP: statistical analysis, data interpretation, manuscript preparation, literature search, and funds collection. MJ: study design and data interpretation. MH: molecular investigations. RC: statistical analysis and data collection. WB and IJ: manuscript preparation and literature search. BL-R and AC: manuscript preparation, literature search, and funds collection. BJ: study design and data collection. DZ: study design.

### Conflict of Interest

The authors declare that the research was conducted in the absence of any commercial or financial relationships that could be construed as a potential conflict of interest.
